# Suppressive Effect of Autocrine FGF21 on Autophagy-Deficient Hepatic Tumorigenesis

**DOI:** 10.3389/fonc.2022.832804

**Published:** 2022-03-07

**Authors:** Jinyoung Kim, Soyeon Lee, Myung-Shik Lee

**Affiliations:** ^1^Severance Biomedical Science Institute, Yonsei University College of Medicine, Seoul, South Korea; ^2^Brain Korea 21 PLUS Project for Medical Science, Yonsei University College of Medicine, Seoul, South Korea; ^3^Department of Internal Medicine, Yonsei University College of Medicine, Seoul, South Korea; ^4^Soonchunhyang Institute of Medi-bio Science, Soonchunhyang University, Cheonan, South Korea

**Keywords:** autophagy, FGF21 (fibroblast growth factor 21), hepatic tumor, YAP1/TAZ pathway, ROS (reactive oxygen species)

## Abstract

Mice with hepatocyte-specific deletion of *autophagy-related 7* (*Atg7*^ΔHep^ mice) develop hepatoma, suggesting that autophagy deficiency could be a factor in the initiation of tumorigenesis. We have shown that FGF21 is induced as a ‘mitokine’ when *Atg7* is disrupted in insulin target tissues such as the liver, which could affect systemic metabolism through endocrine activity. Since FGF21 or other endocrine FGF such as FGF19 can affect tumor growth, we hypothesized that FGF21 produced by *Atg7*-knockout (KO) hepatocytes may affect the behavior of *Atg7*-KO hepatoma in an autocrine manner. We, thus, crossed *Atg7*^ΔHep^ mice with systemic *Fgf21*-KO (*Fgf21*^−/−^) mice to generate *Atg7*^ΔHep^*Fgf21*^−/−^ mice. The number and size of hepatoma of *Atg7*^ΔHep^ mice were significantly increased by additional *Fgf21* KO. The proliferation of *Atg7*-KO hepatocyte was significantly increased by *Fgf21* KO. pYAP1/YAP1 representing YAP1 degradation was significantly decreased in the liver of *Atg7*^ΔHep^*Fgf21*^−/−^ mice compared to *Atg7*^ΔHep^*Fgf21*^+/+^ mice. Consistently, expression of YAP1/TAZ downstream genes was significantly increased in the liver of *Atg7*^ΔHep^*Fgf21*^−/−^ mice compared to *Atg7*^ΔHep^*Fgf21*^+/+^ mice, which could explain the increased size of hepatoma in *Atg7*^ΔHep^*Fgf21*^−/−^ mice. Accumulation of ROS and ROS-mediated DNA damage were increased in the liver of *Atg7*^ΔHep^*Fgf21*^+/+^ mice, which was further aggravated by additional *Fgf21* KO probably due to the absence of positive effect of FGF21 on mitochondrial function, explaining the increased number of hepatoma in *Atg7*^ΔHep^*Fgf21*^−/−^ mice compared to *Atg7*^ΔHep^*Fgf21*^+/+^ mice. These results show that FGF21 produced by autophagy-deficient hepatocytes could have autocrine or paracrine effects on the number and proliferation of autophagy-deficient hepatoma, suggesting that hormones or factors released from autophagy-deficient tumors can influence the behavior or prognosis of the tumor in addition to the effects on host metabolism.

## Introduction

Macroautophagy/autophagy as a cellular process involving lysosomal degradation of the own material of the cell through autophagosome formation (1) is critical for the maintenance of organelle function and metabolic homeostasis (2). Because of such critical function of autophagy, dysregulated autophagy has been incriminated as a pathogenic factor in the development of diverse diseases such as neurodegenerative diseases, metabolic diseases, cardiovascular disease, and cancer.

Regarding the relationship between cancer and autophagy, the crucial roles of autophagy in the cellular response to nutritional deficiency, which is commonly encountered in cancer, indicate the pro-cancer effect of autophagy (3). On the other hand, autophagy suppresses tumor initiation through inhibition of reactive oxygen species (ROS) generation or DNA damage inducing chromosome stability (2, 4). Furthermore, several autophagy genes act as tumor suppressor genes (5–7). Thus, the role of autophagy in cancer will be different according to the stage of carcinogenesis, cellular context, and microenvironment of the tumor. *In vivo* pieces of evidence of suppression of cancer by autophagy include the development of tumor or cancer in autophagy-deficient tissues or animals. Thus, a liver tumor develops in mice with tissue-specific or systemic mosaic deletion of *autophagy-related 7* (*Atg7)* or *Atg5*, critical autophagy genes (8, 9), and the role of autophagy in the initiation or development of hepatocellular carcinoma has been an attractive issue appealing to the research interest of a number of investigators. Regarding the molecular mechanisms of tumor development in autophagy-deficient liver, the roles of genomic damage, accumulation of SQSTM1/p62, an autophagy receptor, inducing activation of KEAP1–NRF2 axis and stabilization of yes-associated protein 1 (YAP1), an effector of the Hippo pathway, have been reported (8–10).

We previously reported that fibroblast growth factor 21 (FGF21) is induced as an integrated stress response or ‘mitokine’ in autophagy-deficient liver tissue in an ATF4-dependent manner, leading to resistance to diet-induced obesity and insulin resistance through endocrine action (11). In addition to the metabolic effects, FGF21 or FGF15/19, a related endocrine FGF, can exert both proliferative and anti-proliferative effects on target cells through FGF receptors (FGFR1-4) (12–15). FGF21 may be able to affect the proliferation of hepatocellular carcinoma cells, as lack of FGF21 has been reported to promote liver cancer associated with metabolic stress such as long-term obesogenic diet feeding or nonalcoholic steatohepatitis (16, 17). Since obesity or metabolic stress can affect diverse aspects of autophagy or lysosomal function (18–21) and cancer tissue could be deficient in autophagy (4, 6–9, 22), FGF21 produced by autophagy-deficient hepatocytes due to genetic or metabolic causes may affect the behavior of hepatoma with autophagy insufficiency. Thus, it could be worthwhile to study the effect of autocrine FGF21 induced by autophagy-deficient hepatocytes on the tumor arising from autophagy-deficient hepatocytes, which could be of clinical value for understanding the behavior of autophagy-deficient cancer and its management. Employing mice with hepatocyte-specific deletion of *Atg7*, we observed that FGF21 produced by autophagy-deficient hepatocytes inhibits growth and proliferation of autophagy-deficient tumor, suggesting a potential therapeutic role of FGF21 or other factors released from autophagy-deficient tumor in the management of cancer.

## Materials and Methods

### Animals

*Fgf21*^−/−^ mice with a targeted disruption of *Atg7* in hepatocyte were generated by crossing Alb-*Cre* mice with *Atg7*^F/F^ mice and then with *Fgf21*^−/−^ mice (*Atg7*^ΔHep^*Fgf21*^−/−^ mice). All animals were maintained in a specific pathogen free (SPF) facility accredited by the Association for the Assessment and Accreditation of Laboratory Animal Care International (AAALAC). All animal experiments were approved by the Institutional Animal Care and Use Committee of Yonsei University Health System (IACUC of YUHS) and were conducted in accordance with the Public Health Service Policy on Humane Care and Use of Laboratory Animals. Nonfasting blood glucose level was measured using an Accu-Chek glucometer (Lifescan) weekly up to 40 weeks of age.

### Glucose Tolerance Test (GTT)

GTT was performed by an intraperitoneal injection of glucose (1 g/kg weight) into overnight-fasted mice. Blood glucose level was measured at 0, 15, 30, 60, and 120 min using an Accu-Chek glucometer (Lifescan) after glucose injection.

### Measurement of Tumor Volume

The greatest longitudinal diameter (length) and the greatest transverse diameter (width) of hepatic tumor from 9-month-old male mice were determined. Tumor volume was calculated by a modified method of ellipsoidal formula: V = (Length × Width^2^)/2 (23).

### Cell Proliferation

Liver tissues were harvested and immediately fixed in 10% neutral buffered formalin (Sigma-Aldrich, HT501320) to make paraffin-embedded blocks. Immunohistochemistry was conducted using paraffin-embedded liver sections with antibody against Ki67 (Abcam, ab15580, 1:200). The percentage of Ki67^+^ hepatocytes among total hepatocytes was determined in more than 20 randomly chosen fields per group by manual counting under BX43 light microscope (Olympus).

### TUNEL Staining

Deparaffinized liver sections were incubated with TUNEL reagent (Roche Applied Science, 11 684 795 910) and diaminobenzidine tetrahydrochloride (DAB) (Dako, K5007) as the color substrate. The percentage of TUNEL^+^ hepatocytes among total hepatocytes was determined in more than 20 randomly chosen fields per group by manual counting under a BX43 light microscope (Olympus).

### Quantitative Real-Time PCR

cDNA was synthesized using total RNA extracted from liver tissue with TRIzol (Thermo Fisher Scientific, 15596018) and M-MLV Reverse Transcriptase (Promega, M17013) according to the protocol of the manufacturer. Real-time RT-PCR was performed using SYBR master mix (Takara, RR420A) in a QuantStudio3 Real-Time PCR System (Applied Biosystems). All expression values were normalized to *Rpl32* mRNA level. The sequences of primers used for real-time RT-PCR are listed in **Supplementary Table S1**.

### Immunoblot Analysis

Liver tissues were lysed with a radioimmunoprecipitation assay buffer (0.1% SDS, 150 mM NaCl, 50 mM Tris, pH 8.0, 1% Triton X-100, 0.5% sodium deoxycholate) containing protease and phosphatase inhibitors. Protein concentration was determined using the Bradford method. Samples (5–10 μg) were separated on 4–12% Bis-Tris gel (NuPAGE^®^, Life Technologies, NP0323), and transferred to nitrocellulose membranes (Merk Millipore, HATF00010) for immunoblot analysis employing the enhanced chemiluminescence (ECL) method (Dongin LS, ECL-PS250). Antibodies against the following proteins used: ATG7 (Cell Signaling Technology, 2631, 1:1,000), FGF21 (R&D Systems, AF3057, 1:1,000), p-FRS2α (Tyr196) (Cell Signaling Technology, 3864, 1:1,000), FRS2 (Santa Cruz Biotechnology, sc-8318, 1:1,000), p-ERK (Thr202/Tyr204) (Cell Signaling Technology, 4370, 1:1,000), ERK (Cell Signaling Technology, 4695, 1:2,000), p-AKT (Ser473) (Cell Signaling Technology, 9271, 1:1,000), AKT (Cell Signaling Technology, 9272, 1:2,000), p-YAP1 (Ser127) (Cell Signaling Technology, 4911, 1:1,000), YAP1/TAZ (Cell Signaling Technology, 8418, 1:1,000), β-actin (ACTB) (Santa Cruz Biotechnology, sc-47778, 1:4,000) or HSP90 (Santa Cruz Biotechnology, sc-13119, 1:2,000). For evaluation of YAP1 dephosphorylation, densitometry of the protein bands was performed using ImageJ software (NIH).

### ROS and DNA Damage

Immunohistochemistry was conducted using deparaffinized liver sections with antibodies against nitrotyrosine (Merk Millipore, 06-284, 1:200), phospho-histone H2A.X (Ser139) (Cell Signaling Technology, 9718, 1:100) or 8-hydroxyguanosine (8-oxoG) (Abcam, ab62623, 1:150). The percentage of nitrotyrosine^+^ area per total area was determined by using a BX43 light microscope (Olympus) and ImageJ software (NIH). The percentage of H2A.X^+^ or 8-oxoG^+^ hepatocytes among total hepatocytes was determined by manual counting under a BX43 light microscope (Olympus). All measurements were conducted in more than 25 randomly chosen fields per group.

For dihydroethidium (DHE) staining, fresh-frozen liver sections were incubated with 10 μM DHE solution (Invitrogen, D23107) for 30 min at 37°C with light protection, followed by confocal microscopy after DAPI staining. Fluorescence imaging was conducted using an LSM780 confocal microscope (Carl Zeiss). The total fluorescence of DHE^+^ cells per field was determined in more than 25 randomly chosen fields per group by using ImageJ software (NIH).

### Cytochrome *c* Oxidase (COX) Staining

For staining of mitochondrial COX activity, unfixed fresh-frozen liver sections were incubated in a reaction buffer containing 20 mg of cytochrome *c*, 20 mg of DAB, 18 ml of sodium phosphate buffer (0.1 M, pH 7.4), and 2 ml of catalase (20 μg/ml). COX activity was visualized with a DAB reaction, and COX^+^ optical density per area was determined in more than 30 randomly chosen fields per group using a BX43 light microscope (Olympus) and ImageJ software (NIH).

### Blood Chemistry

Serum alanine aminotransferase (ALT) and aspartate aminotransferase (AST) levels were measured with a Fuji Dri-Chem NX500 biochemistry analyzer (Fujifilm).

### Statistical Analysis

All values are expressed as means ± SEM. Statistical significance was tested with unpaired two-tailed Student’s *t*-test to compare two groups, and one-way analysis of variance (ANOVA) with Tukey’s test or two-way ANOVA with Bonferroni’s test to compare multiple groups. All analyses were performed using Prism Version 6 software (GraphPad). *P*-values of less than 0.05 were considered to indicate statistically significant differences.

## Results

### Increased Volume and Number of Autophagy-Deficient Hepatic Tumor by *Fgf21* KO

Since FGF21 is produced by *Atg7*-knockout (KO) hepatocytes and FGF21 could have significant impacts on metabolic profile, we first studied metabolic features of *Atg7*^ΔHep^*Fgf21*^+/+^ and *Atg7*^ΔHep^*Fgf21*^−/−^ mice that were generated by crossing *Atg7*^ΔHep^*Fgf21*^+/+^ mice with *Atg7*^F/F^*Fgf21*^−/−^ mice. The absence of *Atg7* and *Fgf21* expression was confirmed by immunoblot analysis using specific antibodies and real-time RT-PCR using specific primers (**Supplementary Figures S1A–C**). Nonfasting blood glucose level was not different between *Atg7*^F/F^*Fgf21*^+/+^, *Atg7*^F/F^*Fgf21*^−/−^, *Atg7*^ΔHep^*Fgf21*^+/+^ and *Atg7*^ΔHep^*Fgf21*^−/−^ mice up to 40 weeks of age on normal chow diet (**Supplementary Figure S1D**). The area under the curve (AUC) of GTT curves was reduced in *Atg7*^ΔHep^*Fgf21*^+/+^ mice on chow diet compared to *Atg7*^F/F^*Fgf21*^+/+^ mice on the same diet (**Supplementary Figure S1E**), which indicates increased glucose clearance, consistent with a previous paper (11). Reduced AUC of GTT curves in *Atg7*^ΔHep^*Fgf21*^+/+^ mice on chow diet was abrogated by additional KO of *Fgf21*, consistent with a previous paper showing the effect of FGF21 release from *Atg7*-deficient insulin target tissues enhancing glucose tolerance, particularly in high-fat diet fed condition (11) (**Supplementary Figure S1E**). AUC of GTT curves was increased in *Atg7*^F/F^*Fgf21*^−/−^ mice compared to *Atg7*^F/F^*Fgf21*^+/+^ mice on the same diet (**Supplementary Figure S1E**), consistent with a previous paper (24).

**Figure 1:**
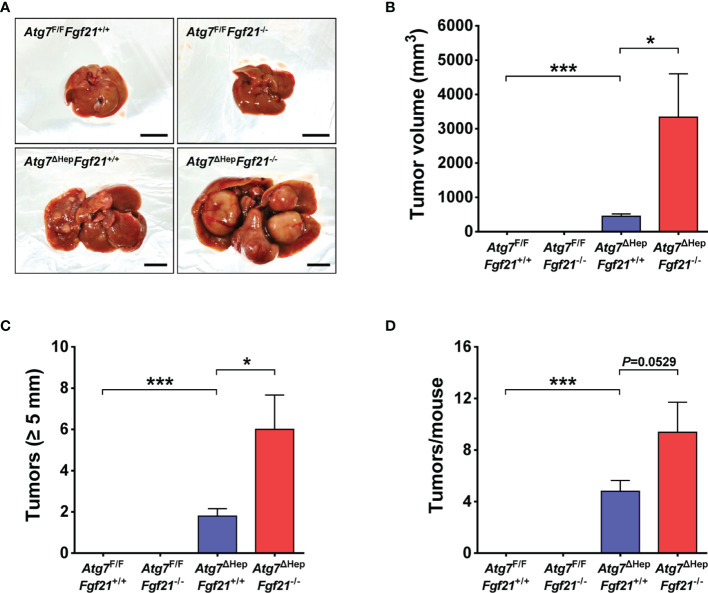
Increased size and number of *Atg7*-KO hepatic tumor by *Fgf21* KO. **(A)** Gross images of the liver and hepatic tumor in *Atg7*^F/F^*Fgf21*^+/+^, *Atg7*^F/F^*Fgf21*^−/−^, *Atg7*^ΔHep^*Fgf21*^+/+^ and *Atg7*^ΔHep^*Fgf21*^−/−^ mice (scale bar, 10 mm). **(B)** Total volume of hepatic tumor in *Atg7*^F/F^*Fgf21*^+/+^, *Atg7*^F/F^*Fgf21*^−/−^, *Atg7*^ΔHep^*Fgf21*^+/+^ and *Atg7*^ΔHep^*Fgf21*^−/−^ mice (*n* = 8–11). **(C)** The number of hepatic tumor with maximal diameter of ≥5 mm in the liver of *Atg7*^F/F^*Fgf21*^+/+^, *Atg7*^F/F^*Fgf21*^−/−^, *Atg7*^ΔHep^*Fgf21*^+/+^ and *Atg7*^ΔHep^*Fgf21*^−/−^ mice (*n* = 8–11). **(D)** Total number of hepatic tumor in the liver of *Atg7*^F/F^*Fgf21*^+/+^, *Atg7*^F/F^*Fgf21*^−/−^, *Atg7*^ΔHep^*Fgf21*^+/+^ and *Atg7*^ΔHep^*Fgf21*^−/−^ mice (*n* = 8–11). All data are shown as means ± SEM. *P <0.05 and ***P <0.001 by one-way ANOVA with Tukey’s test.

As previously reported, *Atg7*^ΔHep^*Fgf21*^+/+^ mice develop liver tumor from 30 weeks of age (**Figure 1A**). Intriguingly, in *Atg7*^ΔHep^*Fgf21*^−/−^ mice, the total volume of the liver tumor was significantly increased compared to that of *Atg7*^ΔHep^*Fgf21*^+/+^ mice (**Figure 1B**). When we classified the liver tumor according to the size of the tumor, the number of liver tumor with a maximal diameter of ≥5 mm which represents most of the total tumor volume was significantly increased in *Atg7*^ΔHep^*Fgf21*^−/−^ mice compared to *Atg7*^ΔHep^*Fgf21*^+/+^ mice (**Figure 1C**). The total number of liver tumors in *Atg7*^ΔHep^*Fgf21*^−/−^ mice also appeared to be increased compared to *Atg7*^ΔHep^*Fgf21*^+/+^ mice, while statistical significance was marginal (**Figure 1D**).

**Figure 2:**
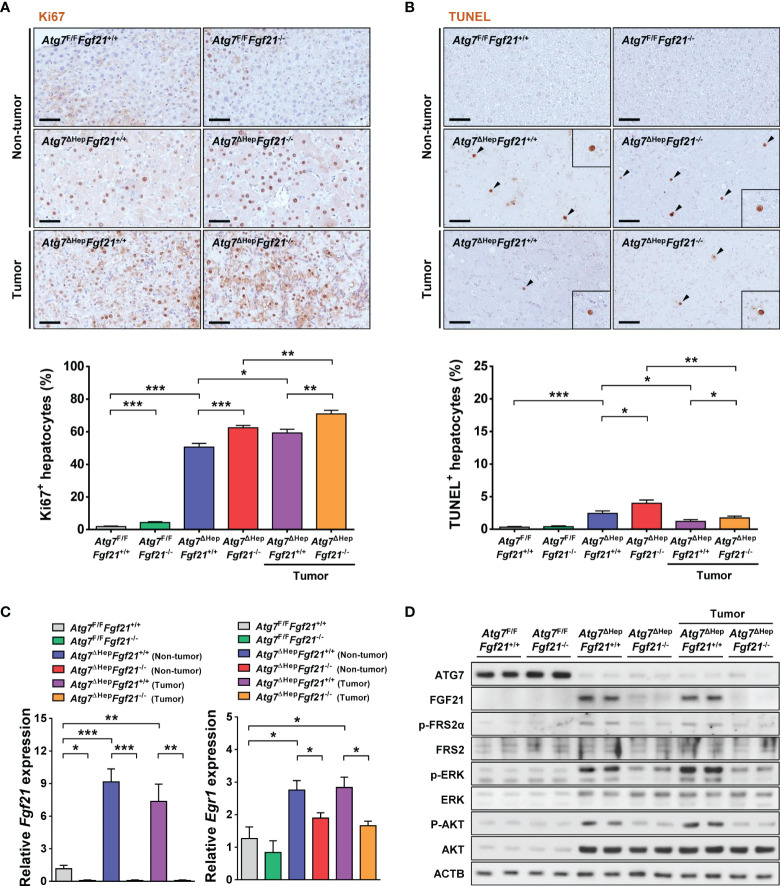
Proliferation and death of autophagy-deficient hepatocytes. **(A)** The percentage of Ki67^+^ cells among total cells was determined after immunohistochemistry using anti-Ki67 antibody (lower). Representative immunohistochemistry is shown (upper) (*n* = 10; scale bar, 50 μm). **(B)** The percentage of TUNEL^+^ apoptotic cells (black arrow heads) among total cells was determined after TUNEL staining (lower). Representative staining is shown (upper) (*n* = 10; scale bar, 50 μm). **(C)** Expression level of *Fgf21* (left) and *Egr1* (right) was examined using real-time RT-PCR in the liver tissues of *Atg7*^F/F^*Fgf21*^+/+^, *Atg7*^F/F^*Fgf21*^−/−^, *Atg7*^ΔHep^*Fgf21*^+/+^ and *Atg7*^ΔHep^*Fgf21*^−/−^ mice. **(D)** Homogenized liver tissues of *Atg7*^F/F^*Fgf21*^+/+^, *Atg7*^F/F^*Fgf21*^−/−^, *Atg7*^ΔHep^*Fgf21*^+/+^ and *Atg7*^ΔHep^*Fgf21*^−/−^ mice were subjected to immunoblot analysis using the indicated antibodies. All data are shown as means ± SEM. *P <0.05, **P <0.01 and ***P <0.001 by one-way ANOVA with Tukey’s test.

Since the increased volume of hepatoma in *Atg7*^ΔHep^*Fgf21*^−/−^ mice compared to *Atg7*^ΔHep^*Fgf21*^+/+^ mice suggested increased proliferation of autophagy-deficient tumor due to the absence of FGF21, we next studied whether the proliferation of autophagy-deficient tumor in *Atg7*^ΔHep^ mice was affected by FGF21. The percentage of Ki67^+^ proliferating cells in the liver of *Atg7*^ΔHep^*Fgf21*^+/+^ mice was significantly increased compared to *Atg7*^F/F^*Fgf21*^+/+^ mice in both non-tumorous and tumorous part of the liver, and that in the tumorous part was slightly but significantly increased compared to that in the non-tumorous part (**Figure 2A**). In the liver of *Atg7*^ΔHep^*Fgf21*^−/−^ mice, the percentage of Ki67^+^ proliferating cells was further significantly increased compared to *Atg7*^ΔHep^*Fgf21*^+/+^ mice in both non-tumorous and tumorous part (**Figure 2A**), which could contribute to the increased size of hepatoma in the liver of *Atg7*^ΔHep^*Fgf21*^−/−^ mice compared to *Atg7*^ΔHep^*Fgf21*^+/+^ mice. The number of TUNEL^+^ cells in the liver of *Atg7*^ΔHep^*Fgf21*^−/−^ mice was slightly but significantly higher than that in the liver of *Atg7*^ΔHep^*Fgf21*^+/+^ mice in both non-tumorous and tumorous parts (**Figure 2B**), indicating increased turnover of *Atg7*-KO hepatocytes by additional KO of *Fgf21*. Serum ALT and AST levels were also significantly increased in *Atg7*^ΔHep^*Fgf21*^−/−^ mice compared to *Atg7*^ΔHep^*Fgf21*^+/+^ mice, consistent with an increased number of TUNEL^+^ cells by additional KO of *Fgf21* (**Supplementary Figure S1F**). Expression of *Fgf21* was significantly increased in both non-tumorous and tumorous parts of the liver of *Atg7*^ΔHep^*Fgf21*^+/+^ mice, and absent in that of *Atg7*^ΔHep^*Fgf21*^−/−^ mice, as expected (**Figure 2C**). Phosphorylation of FRS2α, ERK, and AKT, downstream events of FGF21 action (11, 25), was markedly increased in both non-tumorous and tumorous parts of the liver of *Atg7*^ΔHep^*Fgf21*^+/+^ mice and was abrogated in the liver of *Atg7*^ΔHep^*Fgf21*^−/−^ mice (**Figure 2D**). Expression of *Egr1*, downstream of FGF21-ERK (11), was also increased in both non-tumorous and tumorous parts of the liver of *Atg7*^ΔHep^*Fgf21*^+/+^ mice, and suppressed by additional KO of *Fgf21* (**Figure 2C**).

### Further Increased YAP1 Signaling of Autophagy-Deficient Hepatic Tumor by *Fgf21* KO

**Figure 3:**
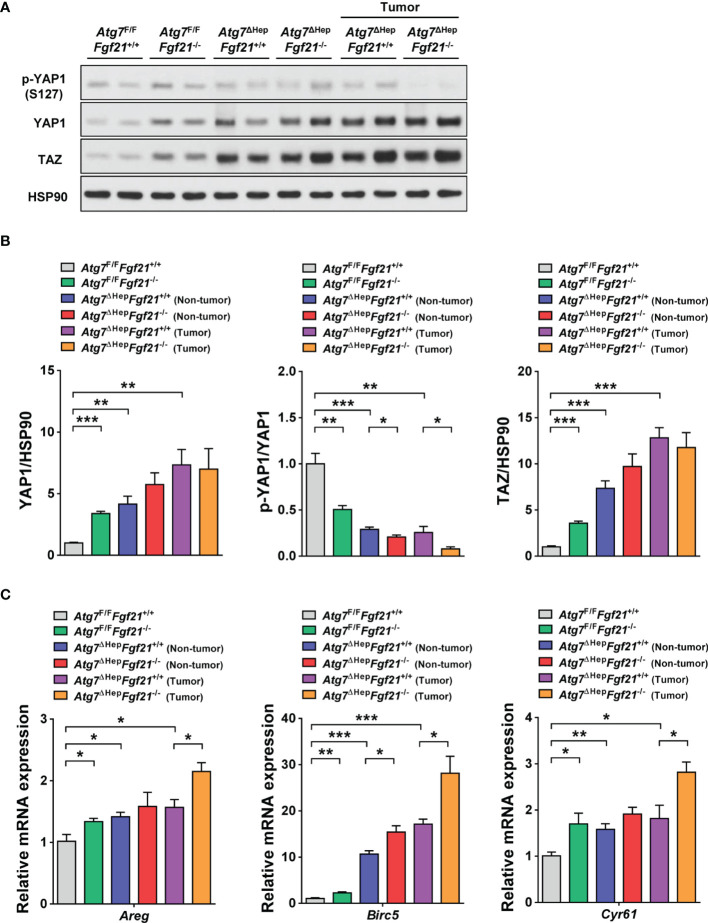
Expression and signaling of YAP1/TAZ in autophagy-deficient hepatocytes. **(A)** Homogenized liver tissues of *Atg7*^F/F^*Fgf21*^+/+^, *Atg7*^F/F^*Fgf21*^−/−^, *Atg7*^ΔHep^*Fgf21*^+/+^ and *Atg7*^ΔHep^*Fgf21*^−/−^ mice were subjected to immunoblot analysis using the indicated antibodies. **(B)** Normalized expression of YAP1 (left) or TAZ (right) and p-YAP1/YAP1 (middle) were calculated after densitometry of immunoblot bands in panel **(A)**. **(C)** Expression level of YAP1 target genes was examined using real-time RT-PCR in the liver tissues of *Atg7*^F/F^*Fgf21*^+/+^, *Atg7*^F/F^*Fgf21*^−/−^, *Atg7*^ΔHep^*Fgf21*^+/+^ and *Atg7*^ΔHep^*Fgf21*^−/−^ mice. All data are shown as means ± SEM. *P <0.05, **P <0.01 and ***P <0.001 by one-way ANOVA with Tukey’s test.

We next studied the mechanism of increased proliferation of autophagy-deficient hepatocytes which was further augmented by additional KO of *Fgf21*. Since a previous paper reported that the degradation of YAP1 by autophagy prevents the development of hepatic tumors (10), we examined the expression level of YAP1. The protein level of YAP1 was significantly increased in the liver of *Atg7*^ΔHep^*Fgf21*^+/+^ mice compared to *Atg7*^F/F^*Fgf21*^+/+^ mice in both non-tumorous and tumorous parts (**Figures 3A, B**), which suggests that impairment of autophagy-dependent clearance of YAP1 (10) can lead to increased proliferation of the liver tissue in *Atg7*^ΔHep^*Fgf21*^+/+^ mice. The protein level of YAP1 was significantly increased in the liver of *Atg7*^F/F^*Fgf21*^−/−^ mice compared to *Atg7*^F/F^*Fgf21*^+/+^ mice (**Figures 3A, B**), which could be due to the ability of FGF receptor such as FGFR4 to induce YAP1 phosphorylation inducing YAP1 degradation (13, 26). While FGFR4 is activated well by FGF15/19 and FGF21 activates FGFR1 most efficiently, FGF21 has also been reported to activate FGFR4 (27, 28). We confirmed the expression of *Fgfr1-4* in the murine liver tissue (**Supplementary Figure S2**), which supports that FGF21 might be able to act through FGFR4. The protein level of YAP1 tended to be increased in the liver of *Atg7*^ΔHep^*Fgf21*^−/−^ mice compared to *Atg7*^ΔHep^*Fgf21*^+/+^ mice in non-tumorous part, while statistical significance was not achieved (**Figures 3A, B**), again likely due to the absence of FGF21-induced YAP1 phosphorylation and degradation. When YAP1 phosphorylation triggering proteasomal degradation of YAP1 (26) was studied, p-YAP1/YAP1 was significantly decreased in both non-tumorous and tumorous parts of *Atg7*^ΔHep^*Fgf21*^+/+^ liver compared to *Atg7*^F/F^ *Fgf21*^+/+^ liver (**Figures 3A, B**), probably due to increased total YAP1 in autophagy-deficient liver tissue of *Atg7*^ΔHep^ mice. p-YAP1/YAP1 was further significantly decreased in both non-tumorous and tumorous part of *Atg7*^ΔHep^*Fgf21*^−/−^ liver compared to *Atg7*^ΔHep^*Fgf21*^+/+^ tumor (**Figures 3A, B**), suggesting that further increased YAP1 due to the absence of FGF21-induced YAP1 phosphorylation and degradation in the same autophagy-deficient background (13) plays a role in the additional increase of tumor size in *Atg7*^ΔHep^*Fgf21*^−/−^ mice compared to *Atg7*^ΔHep^*Fgf21*^+/+^ mice.

Similar to YAP1, the protein level of TAZ was significantly increased in the liver of *Atg7*^ΔHep^*Fgf21*^+/+^ mice compared to *Atg7*^F/F^*Fgf21*^+/+^ mice in both non-tumorous and tumorous parts (**Figures 3A, B**). Again similar to YAP1, the protein level of TAZ was significantly increased in the liver of *Atg7*^F/F^*Fgf21*^−/−^ mice compared to *Atg7*^F/F^*Fgf21*^+/+^ mice (**Figures 3A, B**). The protein level of TAZ also tended to be increased in the liver of *Atg7*^ΔHep^*Fgf21*^−/−^ mice compared to *Atg7*^ΔHep^*Fgf21*^+/+^ mice in non-tumorous part, while statistical significance was not achieved (**Figures 3A, B**). When we studied the expression of YAP1 target genes, expression of *Areg*, *Birc5*, *Cyr61* was significantly increased in the liver of *Atg7*^ΔHep^*Fgf21*^+/+^ mice compared to *Atg7*^F/F^*Fgf21*^+/+^ mice in both non-tumorous and tumorous parts (**Figure 3C**), consistent with the role of YAP1 signaling in the development of liver tumor in *Atg7*^ΔHep^ mice. Their expression was further increased in *Atg7*^ΔHep^*Fgf21*^−/−^ mice compared to *Atg7*^ΔHep^*Fgf21*^+/+^ mice in both non-tumorous and tumorous parts, while statistical significance was achieved only for the tumorous part (**Figure 3C**), which is consistent with the further increased YAP1 signaling in the liver of *Atg7*^ΔHep^*Fgf21*^−/−^ mice compared to *Atg7*^ΔHep^*Fgf21*^+/+^ mice.

### Further Increased ROS-Mediated DNA Damage of Autophagy-Deficient Hepatic Tumor by Additional *Fgf21* KO

**Figure 4:**
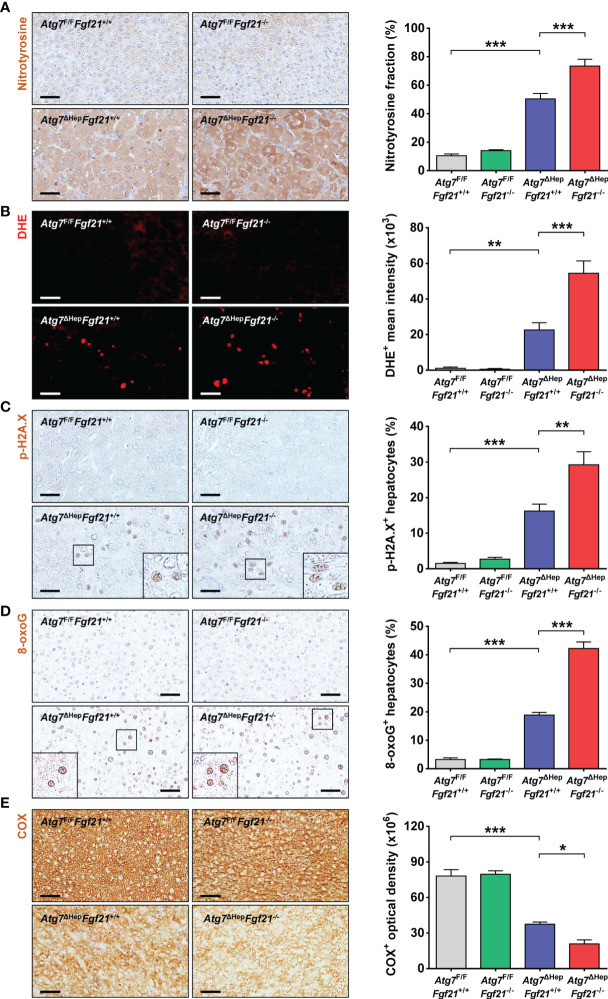
ROS and DNA damage in the liver of autophagy-deficient hepatocytes. **(A)** After immunohistochemistry using anti-nitrotyrosine antibody of the liver sections of *Atg7*^F/F^*Fgf21*^+/+^, *Atg7*^F/F^*Fgf21*^−/−^, *Atg7*^ΔHep^*Fgf21*^+/+^ and *Atg7*^ΔHep^*Fgf21*^−/−^ mice, the proportion of nitrotyrosine^+^ cells among total cells was quantified (right). Representative images are shown (left) (*n* = 5). **(B)** After DHE staining with light protection, the mean fluorescence intensity of DHE^+^ cells was quantified (right). Representative images are shown (left) (*n* = 5). **(C, D)** After immunohistochemistry of the liver sections using p-H2A.X antibody or 8-oxoG antibody, the proportion of p-H2A.X^+^ cells **(C)** or 8-oxoG^+^ cells **(D)** among total cells was quantified (right). Representative images are shown (left) (*n* = 5). **(E)** After staining of mitochondrial COX activity as described in *Materials and Methods*, COX^+^ optical density was quantified (right). Representative images are shown (left) (*n* = 5; scale bar, 50 μm). All data are shown as means ± SEM. *P <0.05, **P <0.01 and ***P <0.001 by one-way ANOVA with Tukey’s test.

Besides the increased proliferation of *Atg7*^ΔHep^*Fgf21*^−/−^ mice compared to *Atg7*^ΔHep^*Fgf21*^+/+^ mice probably due to further increased YAP1 signaling, we studied other possible mechanisms of increased tumorigenicity of *Atg7*^ΔHep^*Fgf21*^−/−^ liver, since an increased number of hepatoma in *Atg7*^ΔHep^*Fgf21*^−/−^ mice compared to *Atg7*^ΔHep^*Fgf21*^+/+^ mice suggested increased initiation of tumor in addition to increased proliferation. Based on the previous paper suggesting the role of ROS-mediated DNA damage in the development of autophagy-deficient tumors (9), we studied ROS accumulation in the liver of *Atg7*^ΔHep^*Fgf21*^−/−^ mice. Content of nitrotyrosine, a marker of oxidative or nitrative stress, was increased in the liver of *Atg7*^ΔHep^*Fgf21*^+/+^ mice compared to *Atg7*^F/F^*Fgf21*^+/+^ mice (**Figure 4A**), probably due to impaired mitochondrial function previously observed in *Atg7*-KO hepatocytes (11). Nitrotyrosine content was further increased in the liver of *Atg7*^ΔHep^*Fgf21*^−/−^ mice compared to *Atg7*^ΔHep^*Fgf21*^+/+^ mice (**Figure 4A**), which could be due to further impaired mitochondrial function in the liver of *Atg7*^ΔHep^*Fgf21*^−/−^ mice compared to *Atg7*^ΔHep^*Fgf21*^+/+^ mice, consistent with previous results reporting the protective effect of FGF21 on mitochondrial function (29, 30). Similarly, DHE fluorescence reflecting ROS accumulation which was increased in the liver of *Atg7*^ΔHep^*Fgf21*^+/+^ mice, was further increased in that of *Atg7*^ΔHep^*Fgf21*^−/−^ mice (**Figure 4B**). We next studied phosphorylation of H2A.X that indicates DNA double-strand breaks or DNA damage response and can explain increased tumorigenesis in autophagy-deficient tissues (31). p-H2A.X was increased in the liver of *Atg7*^ΔHep^*Fgf21*^+/+^ mice compared to *Atg7*^F/F^*Fgf21*^+/+^ mice (**Figure 4C**), likely due to increased ROS. Probably due to further increased ROS, p-H2A.X was again further increased in the liver of *Atg7*^ΔHep^*Fgf21*^−/−^ mice compared to *Atg7*^ΔHep^*Fgf21*^+/+^ mice (**Figure 4C**), suggesting that further increased liver tumor in *Atg7*^ΔHep^*Fgf21*^−/−^ mice could be due to both increased initiation caused by increased ROS and increased proliferation by YAP1 signaling. 8-oxoG, another marker indicating ROS-mediated DNA damage, was similarly increased in the liver of *Atg7*^ΔHep^*Fgf21*^+/+^ mice, which was further increased in that of *Atg7*^ΔHep^*Fgf21*^−/−^ mice (**Figure 4D**). We also evaluated mitochondrial activity in the liver tissue by staining of mitochondrial COX activity (11), since dysfunctional mitochondria, which has been observed in *Atg7*-KO hepatocytes (11), could be the source of ROS production (32–34). ROS could be produced at the mitochondrial complexes I and III by partial inhibition of electron transport (33, 35). As an index of mitochondrial function, COX activity was reduced in the liver of *Atg7*^ΔHep^*Fgf21*^+/+^ mice compared to autophagy-competent liver tissue, indicating impaired mitochondrial activity due to autophagy deficiency. COX activity was further reduced in the liver of *Atg7*^ΔHep^*Fgf21*^−/−^ mice compared to *Atg7*^ΔHep^*Fgf21*^+/+^ mice (**Figure 4E**), suggesting that FGF21 protects mitochondrial function as previously reported (29, 30) and that increased accumulation of ROS and ROS-mediated DNA damage in the liver of *Atg7*^ΔHep^*Fgf21*^−/−^ mice compared to *Atg7*^ΔHep^*Fgf21*^+/+^ mice is probably due to further impaired mitochondrial function caused by additional *Ffg21* KO. Consistently, significantly reduced expression of genes associated with mitochondrial function in the liver of *Atg7*-KO hepatocytes was further downregulated by additional *Fgf21* KO (**Supplementary Figure S3**). The protective effect of FGF21 released from autophagy-deficient hepatocytes as a ‘mitokine’ (11) on mitochondrial function suggests the physiological or adaptive nature of ‘mitokine’ in response to autophagy insufficiency or mitochondrial stress.

## Discussion

We observed that the number and volume of the autophagy-deficient hepatic tumor of *Atg7*^ΔHep^ mice were increased by additional KO of *Fgf21*, which is produced by autophagy-deficient hepatocytes. While the effect of *in vivo* administration of FGF21 on autophagy-deficient or -sufficient hepatocellular carcinoma has not been directly investigated, the role of FGF21 in the development of hepatoma has been demonstrated by the high incidence of nonalcoholic steatohepatitis-associated hepatoma after feeding methionine-deficient high-fat diet or high-fat high-sucrose diet in *Fgf21*-KO mice compared to wild-type mice (16, 17). Furthermore, genetic overexpression of *Fgf21* has been shown to suppress diethylnitrosamine-induced hepatocellular carcinoma (36). In addition, *in vivo* FGF21 administration has been reported to inhibit the growth of prostate cancer which was accompanied by autophagy induction (37). *In vitro* treatment with FGF21 also led to the suppressed proliferation of prostate cancer cells and induction of autophagy (37). These results suggest the suppressive effect of FGF21 on hepatoma and also the possibility that *in vivo* or *in vitro* treatment with FGF21 would exert tumor suppressive effect on autophagy-insufficient hepatoma.

The increase of autophagy-deficient liver tumors was due to both increased initiation and increased proliferation of tumors. Increased initiation of liver tumor in *Atg7*^ΔHep^*Fgf21*^−/−^ mice compared to simple *Atg7*^ΔHep^ mice could be due to further impaired mitochondrial function of autophagy-deficient hepatocytes by additional KO of *Fgf21* inducing further increased ROS damage, which is consistent with previous results reporting the positive effect of FGF21 on mitochondrial function (29, 30). Increased proliferation of liver tumor in *Atg7*^ΔHep^*Fgf21*^−/−^ mice compared to simple *Atg7*^ΔHep^ mice could be due to the absence of FGF21 action on FGF receptor such as FGFR4 inducing phosphorylation and degradation of YAP1 (13, 26) because FGF21 can activate FGFR4, although FGFR4 is activated well by FGF15/19 and FGF21 most efficiently activates FGFR1 (27, 28). These results are in line with previous reports that FGF21 could be a biomarker of hepatic carcinogenesis, particularly that associated with hepatocyte stress (15) and that FGF21 deficiency can promote obesity-induced hepatocellular carcinoma, implying tumor suppressor activity of FGF21 (16). Thus, YAP1 expression in the liver of *Atg7*^ΔHep^*Fgf21*^−/−^ mice was increased by combined effects of both autophagy deficiency and *Fgf21* KO. YAP1, as a member of the Hippo signaling pathway, binds to the promoters of target genes such as *Areg*, *Birc5*, or *Cyr61* in complex with the TEAD transcription factors and would contribute to the initiation or progression of a tumor by regulating cell proliferation, apoptosis susceptibility or cell cycle progression (26, 38). The role of YAP1 in the development of autophagy-deficient tumors is supported by previous results showing suppression of *Atg7*-KO hepatoma by treatment of verteporfin, a YAP1 inhibitor or genetic deletion of *Yap1* (10). YAP1 might also drive c-Myc transcription through interaction with c-Abl, and thereby, enhance hepatoma cell growth (39), while the role of c-Myc in the development of autophagy-deficient hepatoma cells was not studied in this investigation.

**Figure 5:**
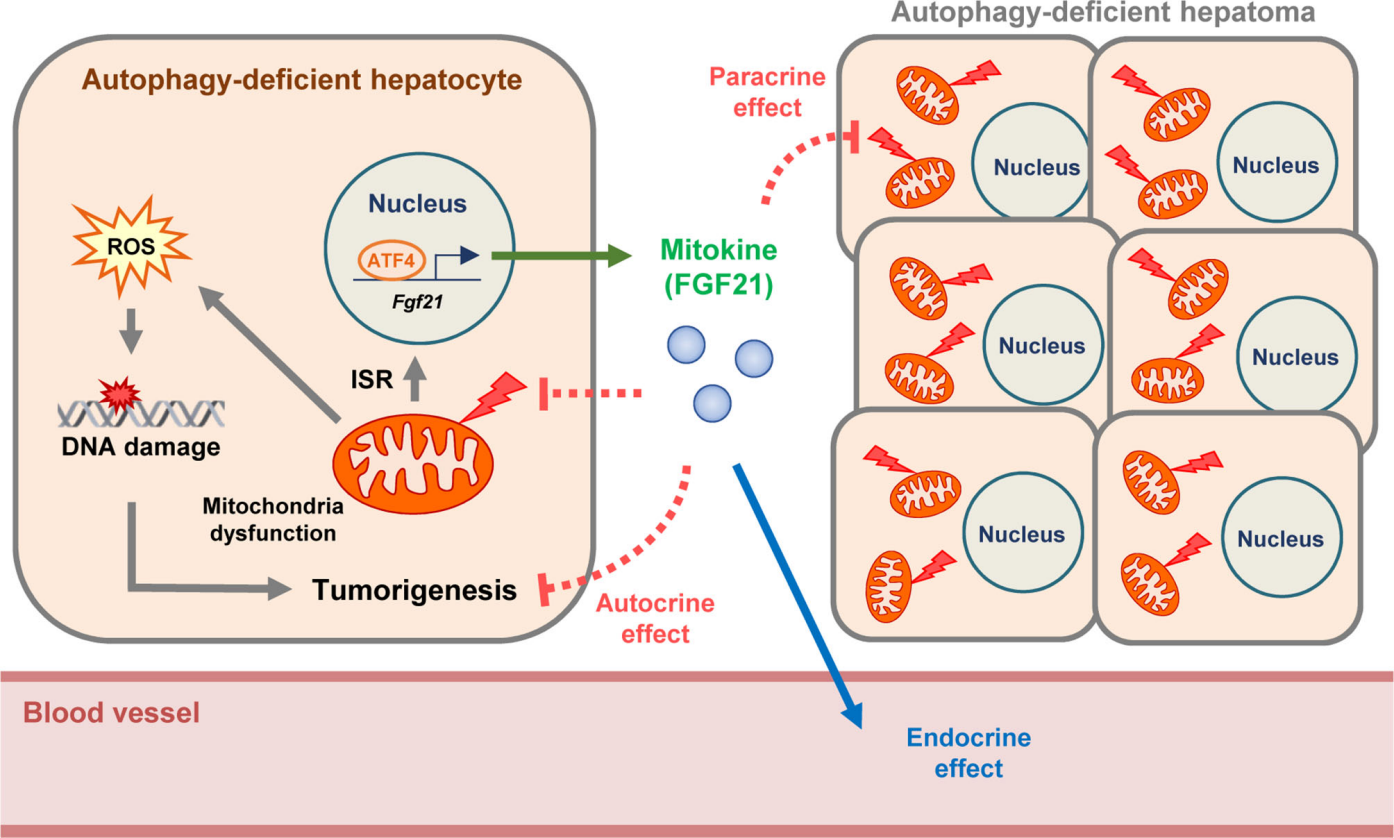
Proposed model of suppressive effect of FGF21 on autophagy-deficient hepatic tumor. FGF21 is produced by autophagy-deficient hepatocytes as an integrated stress response (ISR) and ‘mitokine’, which acts as an endocrine hormone and leads to the amelioration of metabolic stress. We suggest that FGF21 produced by autophagy-deficient hepatocytes with mitochondrial dysfunction suppresses the proliferation of autophagy-deficient hepatocytes and initiation or growth of hepatic tumors in an autocrine or paracrine manner. Moreover, further aggravation of mitochondrial dysfunction of autophagy-deficient hepatocytes by additional KO of *Fgf21* supports protective effect of FGF21 on mitochondrial function and suggests that the ‘mitokine’ response could have a physiological role as an adaptation to autophagy deficiency or mitochondrial dysfunction.

We previously reported that FGF21 is produced by autophagy-deficient hepatocytes as an integrated stress response or ‘mitokine’, which acts as an endocrine hormone and leads to protection against diet-induced obesity and insulin resistance (11). Here, we report that FGF21 produced by autophagy-deficient hepatocytes suppresses the initiation and proliferation or growth of autophagy-deficient hepatocytes or tumors in an autocrine or paracrine manner. Hence, cytokines or factors released from autophagy-deficient cells or tissues could have diverse effects on host metabolism or growth of autophagy-deficient cells themselves through autocrine, paracrine, and endocrine manners (**Figure 5**). Since other hormones such as growth differentiation factor 15 (GDF15) can also be released from autophagy-deficient hepatocytes (Kim et al., unpublished results) and GDF15 can act as either tumor-promoting or tumor-suppressive hormone, several hormones or factors from autophagy-deficient cells or tumors might be able to affect the growth of autophagy-deficient tumors and host metabolism (18, 40, 41). Moreover, further aggravation of mitochondrial dysfunction of autophagy-deficient hepatocytes by additional KO of *Fgf21* supports the protective effect of FGF21, a ‘mitokine’, on mitochondrial function and suggests that the ‘mitokine’ response could have a physiological role as an adaptation to autophagy deficiency or mitochondrial dysfunction. Such effect is similar to the amelioration of mitochondrial dysfunction by other ‘mitokines’ or mitochondria-derived peptides such as humanin or MOTS-c (42, 43) (**Figure 5**). While the mechanism of the protection of mitochondrial function by FGF21 is unclear, enhanced lipid catabolism in stressed conditions might contribute to the attenuation of metabolic stress on mitochondria and maintenance of mitochondrial function (30, 44, 45).

*In vivo* cancer tissue could be either autophagy-sufficient or -deficient depending on the stage of the carcinogenesis and tumor-environmental context (3, 4). Thus, it should be kept in mind that hormones or factors could be released from autophagy-deficient tumors or cancers that can affect the health or metabolic status of the host and prognosis of the patients. For instance, FGF21 can affect food intake, appetite, or food preference (45–47), and GDF15 which can also be released from autophagy-deficient tumors can induce cachexia associated with cancer (48). When certain tumors or cancers are known or suspected to be autophagy-deficient, the search for hormones or factors released from tumor or cancer tissue might be of clinical benefit for the management of the patients. Our data also suggests a potential role of FGF21 or other factors released from autophagy-deficient tumors as therapeutic agents in the management of cancer such as hepatoma.

## Data Availability Statement

The original contributions presented in the study are included in the article/**Supplementary Material**. Further inquiries can be directed to the corresponding author.

## Ethics Statement

The animal study was reviewed and approved by the Institutional Animal Care and Use Committee of Yonsei University Health System (IACUC of YUHS).

## Author Contributions

MSL conceived the paper. JK and SL conducted experiments. JK and MSL wrote the manuscript. All authors listed have made a substantial, direct, and intellectual contribution to the work and approved it for publication.

## Funding

This study was supported by a National Research Foundation of Korea (NRF) grant funded by the Korean government (MSIT) (NRF-2019R1A2C3002924) and by the Bio&Medical Technology Development Program (2017M3A9G7073521). M-SL is the recipient of a grant from the Faculty Research Assistance Program of Yonsei University College of Medicine (6-2016-0055), and the A3 Foresight Program of the NRF (2015K2A2A6002060).

## Conflict of Interest

The authors declare that the research was conducted in the absence of any commercial or financial relationships that could be construed as a potential conflict of interest.

## Publisher’s Note

All claims expressed in this article are solely those of the authors and do not necessarily represent those of their affiliated organizations, or those of the publisher, the editors and the reviewers. Any product that may be evaluated in this article, or claim that may be made by its manufacturer, is not guaranteed or endorsed by the publisher.
