# Supplementary material for: Suppressive Effect of Autocrine FGF21 on Autophagy-Deficient Hepatic Tumorigenesis
Source: Front Oncol. 2022 Mar 7;12:832804. doi: 10.3389/fonc.2022.832804 (PMC8936433; doi:10.3389/fonc.2022.832804)
Supplement: Supplementary file 1 [file DataSheet_1.pdf]

Supplementary Figure S1

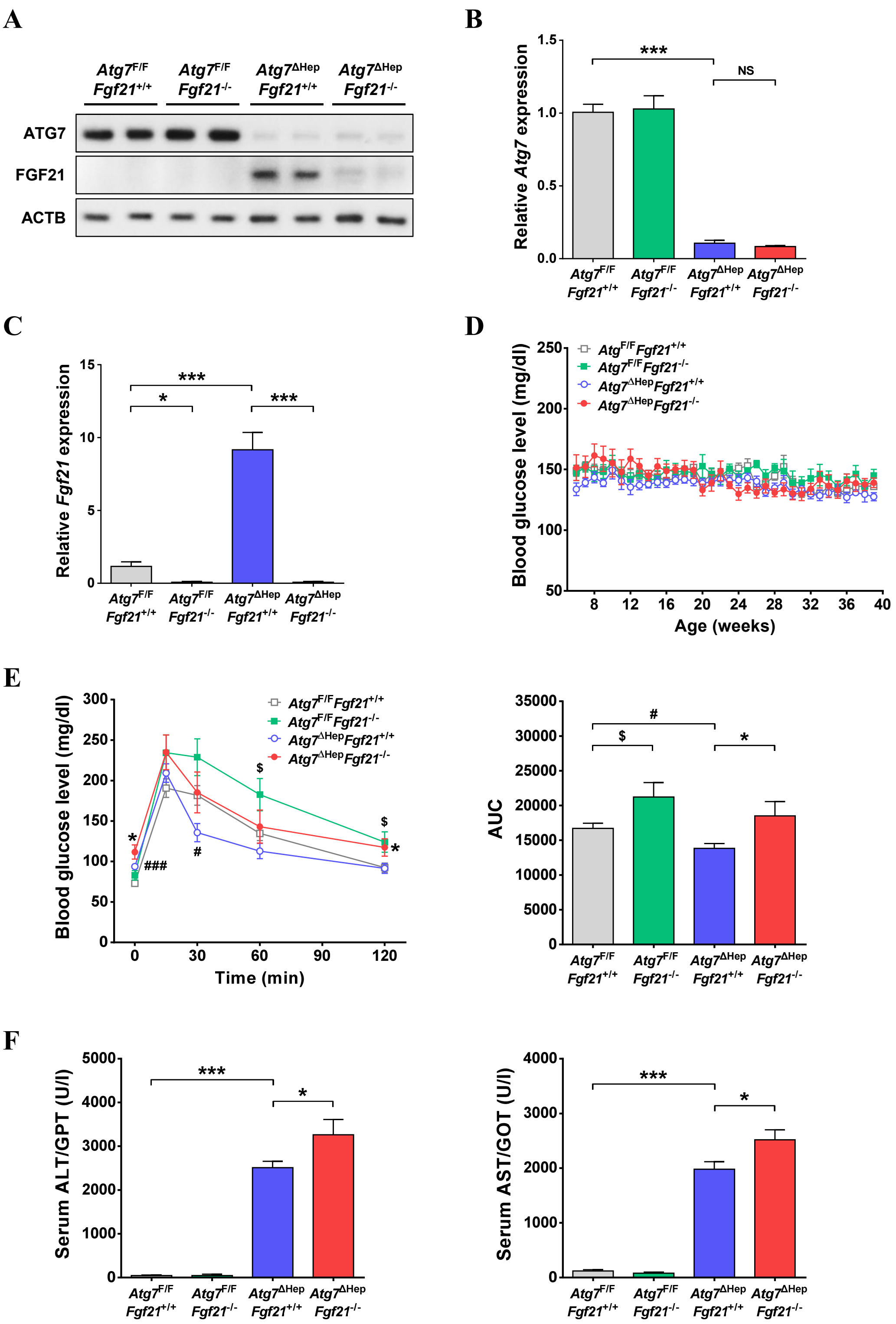

Supplementary Figure S1 | See next page for caption.

Supplementary Figure S1 | Generation and metabolic profile of $Atg7^{\Delta Hep}Fgf21^{-/-}$ mice. **(A)** Immunoblot analysis of the liver tissue from $Atg7^{F/F}Fgf21^{+/+}$, $Atg7^{F/F}Fgf21^{-/-}$, $Atg7^{\Delta Hep}Fgf21^{+/+}$ and $Atg7^{\Delta Hep}Fgf21^{-/-}$ mice using antibodies specific for ATG7, FGF21 or β-actin (ACTB). **(B**, **C)** Relative mRNA expression of *Atg7* and *Fgf21* in the liver tissue from $Atg7^{F/F}Fgf21^{+/+}$, $Atg7^{F/F}Fgf21^{-/-}$, $Atg7^{\Delta Hep}Fgf21^{+/+}$ and $Atg7^{\Delta Hep}Fgf21^{-/-}$ mice. **(D)** Nonfasting blood glucose was monitored weekly up to 40 weeks of age. (n = 8~10) **(E)** Glucose tolerance test (GTT) was conducted in $Atg7^{F/F}Fgf21^{+/+}$, $Atg7^{F/F}Fgf21^{-/-}$, $Atg7^{\Delta Hep}Fgf21^{+/+}$ and $Atg7^{\Delta Hep}Fgf21^{-/-}$ mice (left). Area under the curve (AUC) was calculated (right). (n = 8~10) **(F)** Serum ALT and AST levels in $Atg7^{F/F}Fgf21^{+/+}$, $Atg7^{F/F}Fgf21^{-/-}$, $Atg7^{\Delta Hep}Fgf21^{+/+}$ and $Atg7^{\Delta Hep}Fgf21^{-/-}$ mice. (n = 8~10) All data are shown as means ± SEM. $^{*}P < 0.05$ and $^{***}P < 0.001$ by one-way ANOVA with Tukey's test **(B**, **C**, **E**, **F)** or two-way ANOVA with Bonferroni's test **(D**, **E)**. ($^{\$}$, comparison between $Atg7^{F/F}Fgf21^{+/+}$ and $Atg7^{F/F}Fgf21^{-/-}$ mice; $^{\#}$, comparison between $Atg7^{F/F}Fgf21^{+/+}$ and $Atg7^{\Delta Hep}Fgf21^{+/+}$ mice; *, comparison between $Atg7^{\Delta Hep}Fgf21^{+/+}$ and $Atg7^{\Delta Hep}Fgf21^{-/-}$ mice in **E**). (NS, not significant).

Supplementary Figure S2

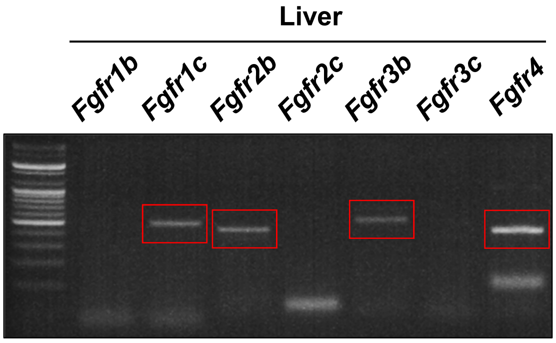

Supplementary Figure S2 | Expression of fibroblast growth factor receptor (FGFR) genes in the mouse liver tissue. RT-PCR was conducted using mRNA prepared from wild-type mouse liver tissue and primers specific for the indicated genes. Rectangles indicate the PCR bands of the correct sizes.

Supplementary Figure S3

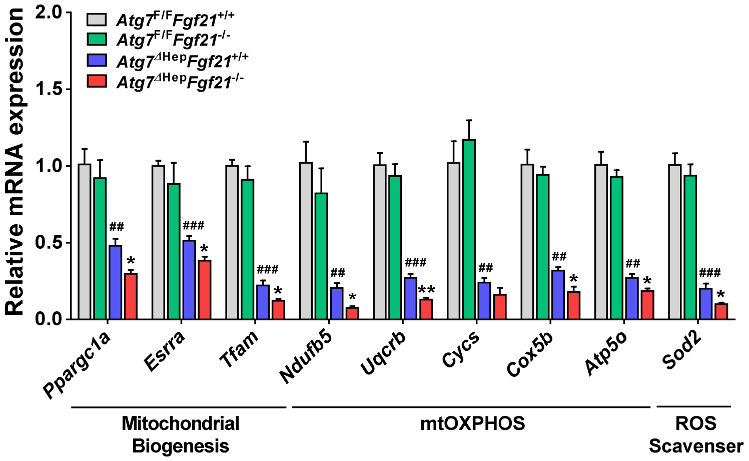

Supplementary Figure S3 | Expression of mitochondrial genes in autophagy-deficient hepatocytes with or without additional KO of *Fgf21*. Relative mRNA expression level of mitochondrial genes in the liver tissue from *Atg7*$^{F/F}$*Fgf21*$^{+/+}$, *Atg7*$^{F/F}$*Fgf21*$^{-/-}$, *Atg7*$^{\Delta Hep}$*Fgf21*$^{+/+}$ and *Atg7*$^{\Delta Hep}$*Fgf21*$^{-/-}$ mice. Data are shown as means ± SEM. $^{*}P < 0.05$, $^{**}P$ or $^{\#\#}P < 0.01$ and $^{\#\#\#}P < 0.001$ by one-way ANOVA with Tukey's test. ($^{\#}$, comparison between *Atg7*$^{F/F}$*Fgf21*$^{+/+}$ and *Atg7*$^{\Delta Hep}$*Fgf21*$^{+/+}$ mice; *, comparison between *Atg7*$^{\Delta Hep}$*Fgf21*$^{+/+}$ and *Atg7*$^{\Delta Hep}$*Fgf21*$^{-/-}$ mice).
