# Supplementary material for: Suppressive Effect of Autocrine FGF21 on Autophagy-Deficient Hepatic Tumorigenesis
Source: Front Oncol. 2022 Mar 7;12:832804. doi: 10.3389/fonc.2022.832804 (PMC8936433; doi:10.3389/fonc.2022.832804)

Figure 2D

IB: ATG7 (Cell Signaling, 2631, 1:1,000)

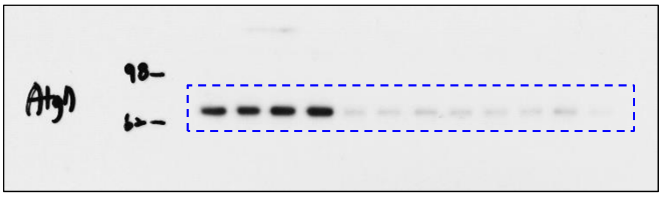

IB: FGF21 (R&D Systems, AF3057, 1:1,000)

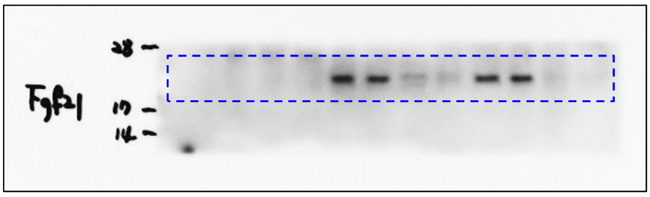

IB: p-FRS2 (Tyr196) (Cell Signaling, 3864, 1:1,000)

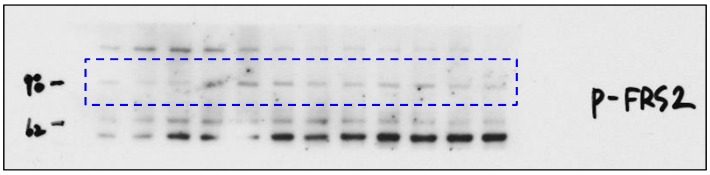

IB: FRS2 (Santa Cruz, sc-8318, 1:1,000)

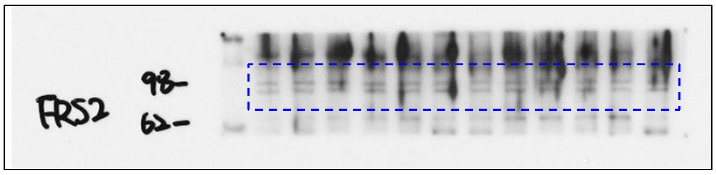

IB: p-ERK (Thr202/Tyr204) (Cell Signaling, 4370, 1:1,000)

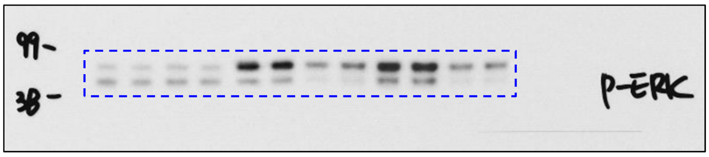

IB: ERK (Cell Signaling, 4695, 1:2,000)

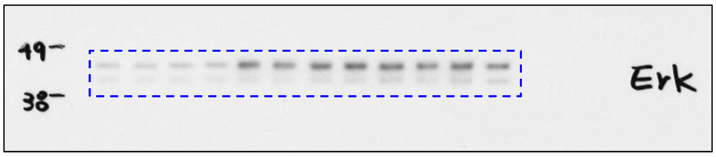

IB: p-AKT (Ser473) (Cell Signaling, 9271, 1:1,000)

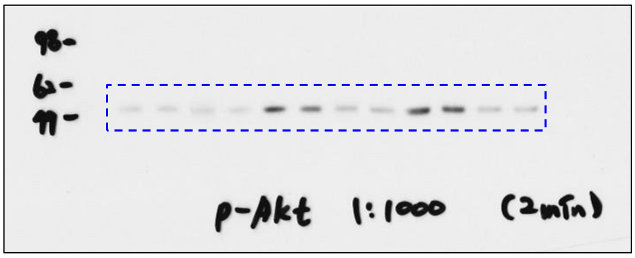

IB: AKT (Cell Signaling, 9272, 1:2,000)

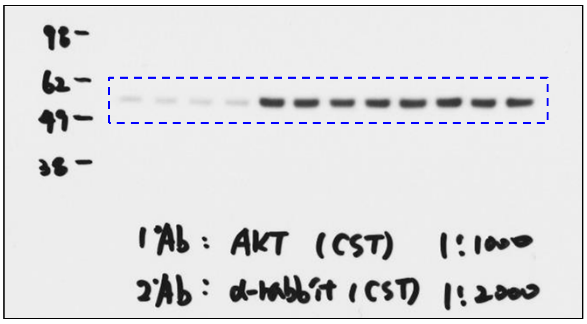

IB: β-actin (ACTB) (Santa Cruz, sc-47778, 1:4,000)

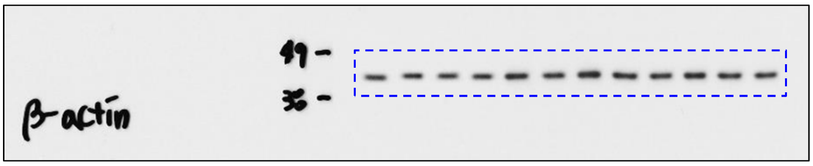

Figure 3A

IB: p-YAP1 (Ser127) (Cell Signaling, 4911, 1:1,000)

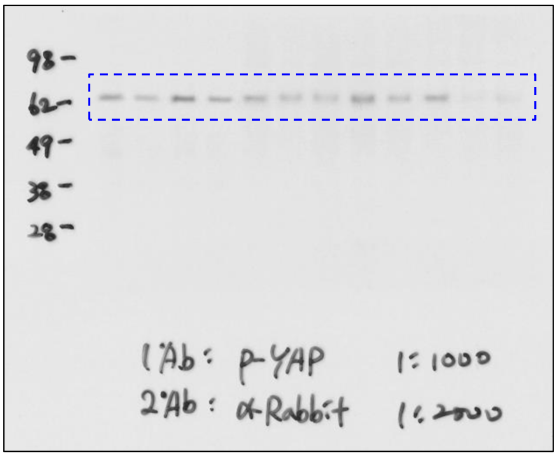

IB: HSP90 (Santa Cruz, sc-13119, 1:2,000)

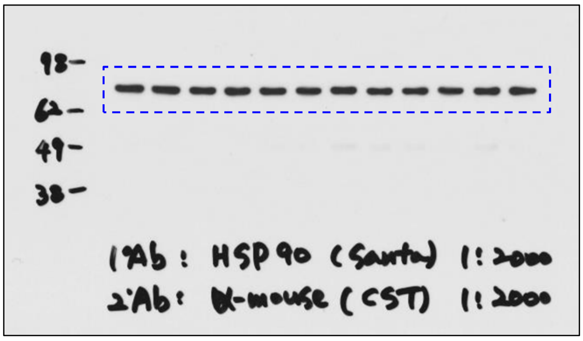

IB: YAP1/TAZ (Cell Signaling, 8418, 1:1,000)

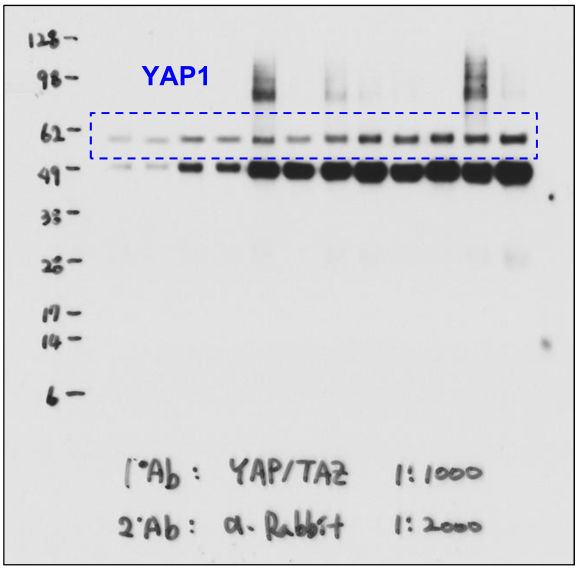

IB: YAP1/TAZ (Cell Signaling, 8418, 1:1,000)

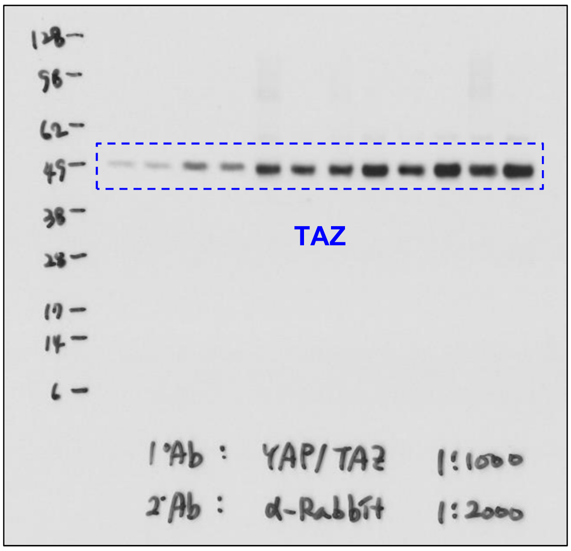

Supplementary Figure S1A

IB: ATG7 (Cell Signaling, 2631, 1:1,000)

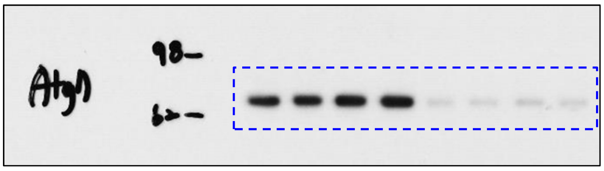

IB: FGF21 (R&D Systems, AF3057, 1:1,000)

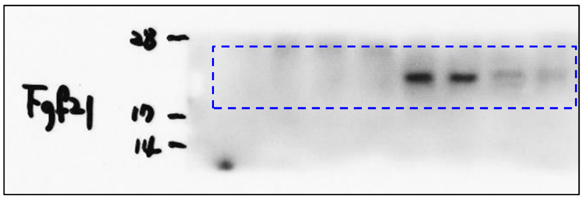

IB: β-actin (ACTB) (Santa Cruz, sc-47778, 1:4,000)

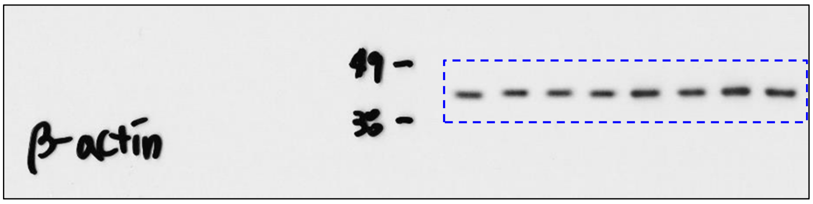

Supplement: Supplementary file 2 [file DataSheet_2.pdf]
