# Supplementary material for: Suppressive Effect of Autocrine FGF21 on Autophagy-Deficient Hepatic Tumorigenesis
Source: Front Oncol. 2022 Mar 7;12:832804. doi: 10.3389/fonc.2022.832804 (PMC8936433; doi:10.3389/fonc.2022.832804)
Supplement: Supplementary file 3 [file Table_1.pdf]

Supplementary Table S1 | Sequences of primers for quantitative RT-PCR.

Gene	Forward (5’ to 3’)	Reverse (5’ to 3’)
Areg	TTTGGTGAACGGTGTGGAGA	GATGCCGATGCCAATAGCTG
Atg7	TGTGGAGCTGATGGTCTCTG	TGATGGAGCAGGGTAAGACC
Atp5o	TCTGGCGCCAGTAGTCTCTT	AGATGATACCCTGGGTGTTG
Birc5	CCCGATGACAACCCGATAGA	GTTGGTCTCCTTTGCAATTTTGT
Cox5b	AGGCAGCTTCAGGCACCAAG	GGTGGGGCACCAGCTTGTAA
Cycs	TACCCACTCAGAAACACACA	GTCTGAAGTCACGATGAGGT
Cyr61	GTGTCAAGAAATACCGGCCC	CTCCATCTTCGCATCGGAAC
Egr1	GAGAACCGTACCCAGCAGCC	CGATCGCAGGACTCGACAGG
Esrra	AGAGACTGAGACTGAACCCC	TTTGGGTAGAGAGCTGAGCA
Fgf21	TACACAGATGACGACCAAGA	GGCTTCAGACTGGTACACAT
Fgfr1b	CAACCTTGCCTGAACAAGCTC	ACAGGCCTACGGTTTGGTTTG
Fgfr1c	CACCAAACCAAACCCTGTAGC	CAAGTTGTCTGGCCCGATCT
Fgfr2b	GGAATCCAACGCCCACAATG	CCGTCACATTGAACAGAGCC
Fgfr2c	GTTGAAAGATGCCGCCGTG	TTTGTCTGACGGGACCACAC
Fgfr3b	TCTGTCGAGCCACCAATTTCA	GGTCCTTGTCAGTCGCATCA
Fgfr3c	GGCAAAGAATTCCGAGGGGA	GCTCCTTGTCGGTGGTGTTA
Fgfr4	TGGCCCTGTTGAGCATCTTT	GTTGGGGGTGTGTCCAGTAG
Ndufb5	TCCTAGACTCGGAGTCGGAA	AACTTCCTGCTCCTTTAACC
Ppargc1a	GCAGCCAAGACTCTGTATGG	CGCTACACCACTTCAATCCA
Rpl32	CAGTCAGACCGATATGTGAA	TAGAGGACACATTGTGAGCA
Sod2	GACCTGCCTTACGACTATGG	GACCTTGCTCCTTATTGAAGC
Tfam	TCGCATCCCCTCGTCTATC	GGGCTGCAATTTTCCTAACC
Uqcrb	ACTTACCCAGAAGGCAGCG	TGCCCACTCTTCTCTCTCCT
